# Quantification of Nanoparticle Enhancement in Polarized Breast Tumor Macrophage Deposits by Spatial Analysis of MRI and Histological Iron Contrast Using Computer Vision

**DOI:** 10.1155/2018/3526438

**Published:** 2018-10-30

**Authors:** Avigdor Leftin, Jason A. Koutcher

**Affiliations:** ^1^Department of Medical Physics, Memorial Sloan Kettering Cancer Center, New York, NY, USA; ^2^Department of Medicine, Memorial Sloan Kettering Cancer Center, New York, NY, USA; ^3^Molecular Pharmacology Program, Memorial Sloan Kettering Cancer Center, New York, NY, USA

## Abstract

Magnetic resonance imaging applications utilizing nanoparticle agents for polarized macrophage detection are conventionally analyzed according to iron-dependent parameters averaged over large regions of interest (ROI). However, contributions from macrophage iron deposits are usually obscured in these analyses due to their lower spatial frequency and smaller population size compared with the bulk of the tumor tissue. We hypothesized that, by addressing MRI and histological pixel contrast heterogeneity using computer vision image analysis approaches rather than statistical ROI distribution averages, we could enhance our ability to characterize deposits of polarized tumor-associated macrophages (TAMs). We tested this approach using *in vivo* iron MRI (FeMRI) and histological detection of macrophage iron in control and ultrasmall superparamagnetic iron oxide (USPIO) enhanced mouse models of breast cancer. Automated spatial profiling of the number and size of iron-containing macrophage deposits according to localized high-iron FeMRI or Prussian blue pixel clustering performed better than using distribution averages to evaluate the effects of contrast agent injections. This analysis was extended to characterize subpixel contributions to the localized FeMRI measurements with histology that confirmed the association of endogenous and nanoparticle-enhanced iron deposits with macrophages in vascular regions and further allowed us to define the polarization status of the macrophage iron deposits detected by MRI. These imaging studies demonstrate that characterization of TAMs in breast cancer models can be improved by focusing on spatial distributions of iron deposits rather than ROI averages and indicate that nanoparticle uptake is dependent on the polarization status of the macrophage populations. These findings have broad implications for nanoparticle-enhanced biomedical imaging especially in cancer.

## 1. Introduction

Widespread efforts have succeeded in integrating nanoparticles in virtually all areas of medical imaging. The appeal of these formulations derives from their ability to be tailored to specific applications ranging from neuroscience to oncology by chemical manipulation of nanoparticle composition rendering them visible to multiple imaging modalities such as MRI, PET, and optical imaging systems [1]. Moreover, the ability to functionalize these particles using delivery systems such as polymers or lipids and bioaffinity tags such as antibodies further enhances our ability to probe, monitor, and control ubiquitous biological processes spanning drug delivery and cell tracking [2].

Nanoparticles formulated from iron are ubiquitous contrast agents for MRI. One common agent used consists of ultrasmall superparamagnetic iron oxide (USPIO) nanoparticles solubilized with dextran polymer to facilitate suspension and delivery in aqueous solution [3, 4]. Cancer research is a premier application of these USPIO nanoparticles, and they have been used to investigate virtually every cancer type. They provide information about both vascular and immune cell properties of the tumor that are key determinants of the pharmacodynamic behavior of drugs and the cellular immune response to therapies [5–7].

Quantification of tissue uptake of iron nanoparticles and deposition in macrophages is conventionally performed using region of interest (ROI) analysis of MRI images [8]. Pixel contrast levels, relaxation times, rates, or the susceptibility phase [9–12] are measured over tissue cross-sectional areas and analyzed according to pixel distribution statistics. Changes in iron concentration following USPIO injection can be further quantified from these pixel distributions according to parametric relations between these observables and known iron standards, iron-labeled cells, or biopsy iron measurement to provide quantitative estimates on nanoparticle delivery, cellular uptake, and concentration [13–16]. So-called iron MRI (FeMRI) approaches can estimate iron nanoparticle uptake by the measurement of parametric distribution statistics. However, it has long been an accepted caveat of the quantitative interpretation of most cellular MRI applications that ROI-based distribution analysis is biased by contributions from the abundant low-iron areas of the tissue, i.e., those not containing the iron deposit or contrast agent, which limits the specificity of the MRI pixel distribution analysis for iron accumulation in rare cellular targets such as macrophages in tumors.

Macrophages are important imaging targets in cancer because they can function in both inflammatory and wound-healing roles that influence tumor growth and therapeutic response [17–19]. Nanoparticle injection and uptake is dependent upon and can influence this polarization status of the targeted cellular populations as these cells exhibit plasticity in these functional roles that in turn is coupled to their innate role in iron metabolism [20, 21]. While MRI studies do not directly report on polarization, immunofluorescence imaging can be used to evaluate changes in TAM iron deposit polarization and provide subpixel information about the dependence of the USPIO contrast agent uptake on TAM polarization [22–24]. However, nanoparticle delivery to the tumor rarely exceeds a few percent of the injected dose [25], and therefore, only a small fraction of macrophages present in the tumor will be engaged by the nanoparticles and give rise to localized detectable iron contrast. Therefore, similar to cellular iron MRI, whole ROI analysis of histological polarization state measurements is more representative of the nanoparticle-free macrophage populations rather than subpopulations containing iron. Therefore, scoring macrophage polarization with immunofluorescence using whole ROI distribution analysis also will generally not be representative of the relatively rare iron-handling populations targeted by the nanoparticle injections. Thus, alternative unbiased analysis approaches that better quantify the local distributions of these iron-containing cells and their phenotypes are required to evaluate the polarization status of the macrophage targets and to provide cellular level corroboration of FeMRI applications.

In the current study, we advance a computer vision approach to localize polarized macrophages according to iron status in order to improve their quantification in USPIO-enhanced cellular imaging by MRI and histology. We have previously shown that, by addressing the spatial heterogeneity of iron-dependent image contrast, we could enhance the quantification of these macrophage deposits without contrast agents using MRI and histology because the type of iron stored in the macrophages generates stronger MRI contrast enhancement compared with venous or hemorrhagic blood [26–30] and Prussian blue only labels macrophage with solid iron deposits corresponding to those of the highest iron concentration. These endogenous stores conferred sufficient cellular sensitivity and specificity to detect macrophage iron deposits in multiple cancer models including prostate cancer and breast cancer, in both primary and distant disseminated metastatic locations of the lung and brain, as well as systemically in organs which naturally or pathologically accumulate macrophage iron such as the liver and spleen [29, 30]. In the current study, we continue our translational development of this approach by combining it with the USPIO contrast agent injection to evaluate iron deposition in polarized breast tumor macrophages.

## 2. Results

**Figure 1:**
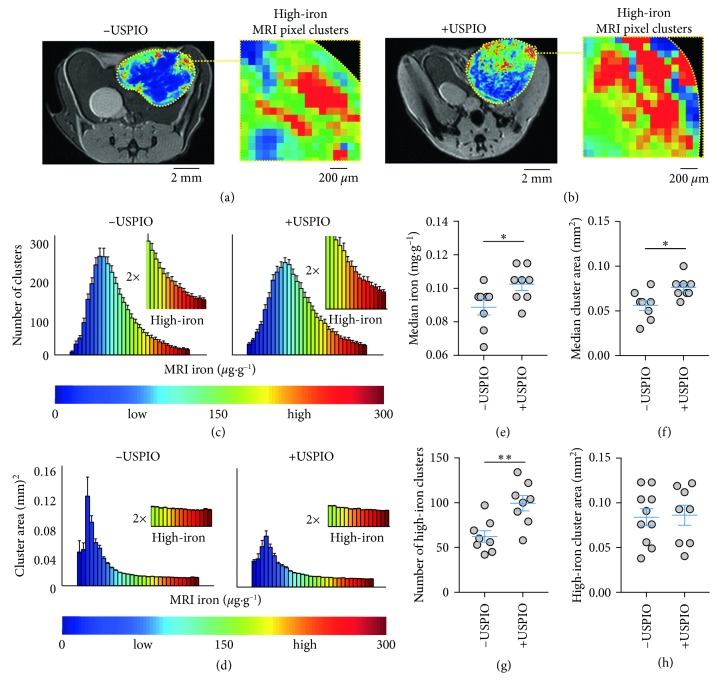
Spatial quantification of endogenous and nanoparticle-enhanced iron deposits with MRI *in vivo*. T_2_-weighted MRI and iron concentration overlay images of (a) control (−USPIO) and (b) iron nanoparticle-injected (+USPIO) tumors. Expansion shows high-iron pixel contrast in clustered areas. (c) Number (#) of clusters and (d) area of the pixel clusters in control (−USPIO) and nanoparticle-injected (+USPIO) tumors as a function of iron concentration. Distributions are from whole cross-sectional regions of interest (ROI) areas of tumors measuring approximately 1 cm^3^ (mean ± SEM shown, *n*=8 tumors/group). MRI iron concentration range at bottom corresponds to values in iron images above. Control (−USPIO) and nanoparticle-injected (+USPIO) (e) median iron concentrations and (f) pixel cluster sizes. (g) Number (#) of high-iron pixel clusters and (h) size of the high-iron clusters from localized computer vision analysis (mean ± SEM shown, *n*=8 tumors/group, n.s. *p* > 0.05, ^*∗*^*p* < 0.05, ^*∗∗∗*^*p* < 0.001, two-tailed unpaired Student's *t*-test). Scale bars are shown for all images.

To demonstrate the difference between the ROI average distribution measurements and the localized measurements of cellular iron deposition with and without dextran-USPIO injection, we initiated orthotopic MMTV-PyMT murine mammary tumors and injected USPIO (0.5 mmol/kg) or saline intravenously once tumor volumes reached approximately 1 cm^3^. Mapping iron-dependent contrast 24 hrs after injection at 7T showed that both the nanoparticle-free (Figure 1, −USPIO) and contrast-enhanced tumors (Figure 1, +USPIO) evidenced heterogeneous distributions of pixel iron levels, with both cohorts exhibiting numerous high-iron pixel regions, i.e., clusters indicative of iron deposits, at the periphery of the tumor, and iron oxide-injections increased the numbers of these clusters.

Analysis of the iron MRI maps was then performed to quantify these pixel distributions. Parametric pixel distributions expressed as number of pixel clusters (Figure 1) and cluster size measured in mm^2^ (Figure 1) were reconstructed as a function of iron concentration with high and low ranges defined with respect to the median iron level of the distribution range. Frequency (number of pixel clusters) and size distribution maxima fell well within the low-iron contrast range in both control and USPIO-injected tumors and appeared to shift nominally towards higher concentrations with USPIO injection. In the high iron range of the frequency distribution, clear increases of pixel clusters occurred with the USPIO uptake, while the size of these high-iron clusters appeared to remain constant in the control and nanoparticle-treated groups suggesting that iron injection changed only the number of the deposits but not their size. Statistical analysis of the ROI-based parametric iron distributions was then performed to quantify the observations. First, median values were calculated from ROI analysis over whole tumor cross sections as is conventionally performed. Median iron levels of the parametric pixel distributions from the ROI showed small but significant increases in frequency (Figure 1; *p* < 0.05) and size (Figure 1; *p* < 0.05) with USPIO enhancement. In order to more specifically quantify local accumulation of iron deposits indicated by the high-iron range stratification, the frequency of high-iron clusters in the maps was counted, and the areas were measured. This revealed significant increases of the high-iron pixel clusters with iron nanoparticle injection (Figure 1; *p* < 0.001) and provided an additional measurement of the size of these regions of iron accumulation with and without contrast agents (Figure 1; *p* > 0.05). This demonstrates an improvement over the ROI quantification in terms of the significant increases in iron deposit accumulation detected and confirms the observation that the number of high-iron clusters increase but the size remains relatively unchanged with USPIO injection.

**Figure 2:**
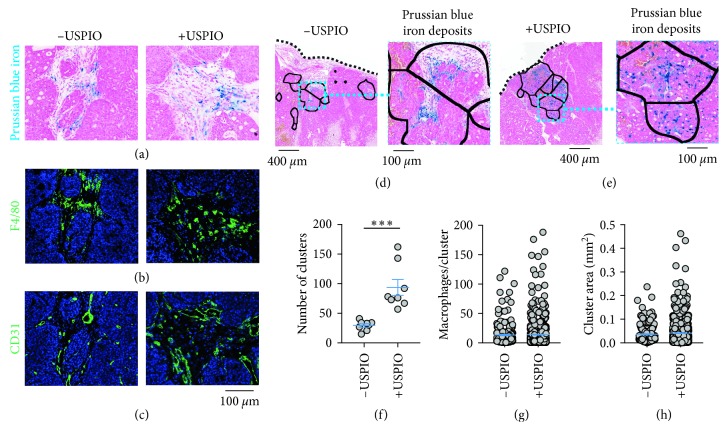
Spatial quantification of endogenous and contrast-enhanced macrophage deposits by iron histology. Paraffin-embedded sections from control (−USPIO) and iron nanoparticle-injected (+USPIO) tumors. (a) Prussian blue iron staining, (b) F4/80 macrophage immunofluorescence, and (c) CD31 vascular immunofluorescence were performed in the same sections to demonstrate the colocalization of iron deposits with infiltrating macrophages in vascular areas of the tumor microenvironment. Histological fields from (d) control and (e) iron nanoparticle-injected mice showing deposits of Prussian blue iron^+^ macrophages localized in infiltrative border regions in discrete clusters. Black borders are regions of interest drawn automatically around deposits of cells according to iron status. Expansion shows iron^+^ macrophages in the deposit regions. (f) Number of iron deposits (clusters) per tumor cross section in control (−USPIO) and nanoparticle-injected (+USPIO) mice (mean ± SEM shown, *n*=8 tumors/group, ^*∗∗∗*^*p* < 0.001, two-tailed unpaired Student's *t*-test). (g) Number of iron^+^ macrophages per deposit, and (h) size of each of the deposits measured over the whole Paraffin-embedded tumor cross sections stained by Prussian blue (mean ± SEM shown, −USPIO *n*=235 total clusters/group, +USPIO *n*=748 total clusters/group, n.s. *p* > 0.05). Scale bar is shown for all images.

To confirm the cellular identity of the iron^+^ species and characterize the microenvironment of the iron deposits, Prussian blue iron histochemistry (Figure 2) and immunofluorescence for F4/80^+^ (Figure 2) macrophages and CD31^+^ vasculature (Figure 2) was performed. Iron staining of the MMTV-PyMT tumor sections evidenced iron^+^ cellular species in stromal regions almost exclusively at peripheral tumor margins. These localized cellular iron sources clustered as multicellular deposits in both the control and USPIO-treated tumors. Macrophage immunofluorescence of these tissue cross sections further confirmed that the iron^+^ cells identified in the Prussian blue histology were colocalized with F4/80^+^ macrophages. These iron^+^ F4/80^+^ macrophages were invariably found in close proximity to CD31^+^ vasculature in both control and USPIO-injected cohorts suggesting that the accumulation of metabolic or nanoparticle-derived iron is dependent upon their spatial distribution beside tumor vasculature.

The spatial characteristics of the histologically detected macrophage iron deposits were then analyzed in a manner similar to the FeMRI pixel cluster analysis. The endogenous (Figure 2) and USPIO-enhanced deposits (Figure 2) found in the localized regions were automatically identified and the number of the clusters (Figure 2), the number of iron^+^ macrophages per deposit (Figure 2), and the areas of the clusters (Figure 2) were measured exhaustively from whole tumor axial cross sections. The number of deposits containing iron^+^ macrophages increased significantly with USPIO injection (*p* < 0.001), and deposit areas in the control and injected groups were found to be equivalent, approximately a few MRI square-pixels (*p* > 0.05). These regions also equivalently contained an average of approximately 14 iron^+^ macrophages per control or USPIO-enhanced deposit (*p* > 0.05) supporting the cellular sensitivity of the FeMRI measurement and specificity of the pixel cluster analysis for these cellular species.

**Figure 3:**
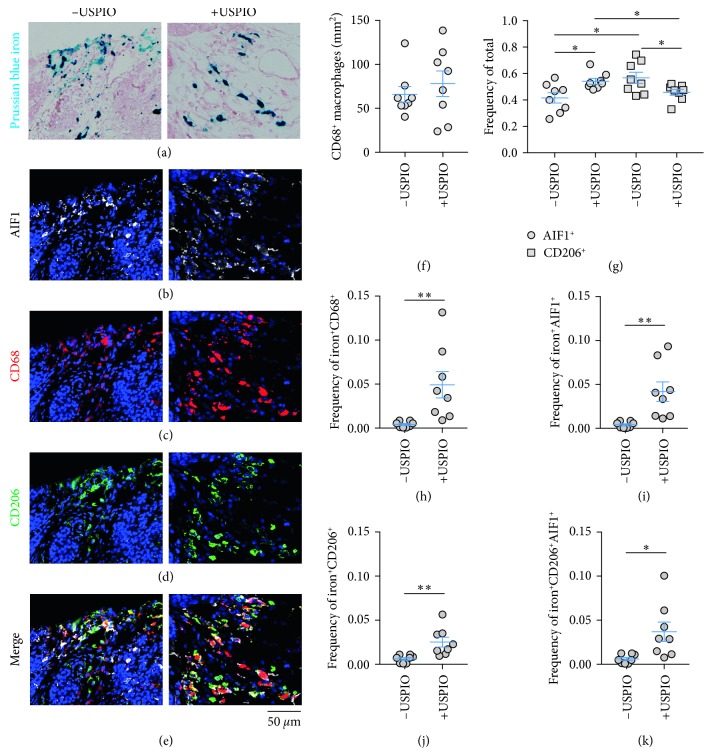
Characterization of endogenous and contrast-enhanced macrophage iron deposit polarization. Fields of paraffin-embedded tumor cross sections from control (left, −USPIO) and iron nanoparticle-injected (right, +USPIO) mice showing deposits of (a) Prussian blue iron^+^ macrophages and colocalized (b) AIF1, (c) CD68, (d) CD206, and (e) merged immunofluorescent markers. (f) Absolute count of CD68^+^ macrophages per mm^2^ tumor cross section and (g) frequency of total M1-like (AIF1) and M2-like (CD206) macrophages determined by whole tumor cross section ROI analysis of immunofluorescent images in control (−USPIO) and nanoparticle-injected (+USPIO) cohorts (mean ± SEM shown, *n*=8 tumors/group, ^*∗*^*p* < 0.05, two-tailed Student's *t*-test). Frequency of total iron^+^ macrophage calculated as (h) iron^+^CD68^+^/CD68^+^, (i) iron^+^AIF1^+^/AIF1^+^, (j) iron^+^CD206^+^/CD206^+^, and (k) iron^+^CD206^+^AIF1^+^/CD206^+^AIF1^+^ cells (mean ± SEM shown, *n*=8 tumors/group, ^*∗*^*p* < 0.05, ^*∗∗*^*p* < 0.01, two-tailed Student's *t*-test). Scale bar is shown for all images.

To determine the polarization status of general macrophage populations in the tumors and their changes with injection of USPIO, immunofluorescence staining of the tumor cross sections was conducted for pan-macrophage (CD68), inflammatory (M1-like AIF1 (allograft inflammatory factor 1)), and wound-healing surface marker phenotypes (M2-like CD206 (mannose receptor)), besides Prussian blue iron histology as the primary observable (Figures 3–3). Absolute counts of CD68^+^ macrophages conducted over whole tissue cross-sectional areas were performed to score CD68 infiltrates (Figure 3; *p* > 0.05). AIF1^+^ and CD206^+^ polarization markers were similarly quantified to evaluate phenotypic shifts. Exhaustive counts performed over the control tumor cross sections and calculation of frequency of these populations with respect to total numbers of these macrophages (M1 or M2/(M1+M2)) (Figure 3) showed that USPIO injection did not lead to significant changes in absolute counts of macrophages (*p* > 0.05). Phenotypic populations in the control mice were significantly biased towards M2-like polarization with 42% M1-like AIF1^+^ and 58% of macrophages CD206^+^ (*p* < 0.05). This analysis of TAM polarization was also performed in the USPIO-injected animals and showed that M1 and M2 status was significantly different with 54% AIF1^+^ and 46% CD206^+^ TAMs present (*p* < 0.05) indicating that USPIO injection caused a significant increase in M1-like cells and reduction of M2-like cells (*p* < 0.05). This demonstrates that TAM polarization and not number of macrophages in the tissue changed as a function of iron nanoparticle injection in these measurements.

We then measured the frequency of specifically iron^+^-polarized TAMs with respect to their respective general population (iron^+^M1^+^ or iron^+^M2^+^/M1^+^ or M2^+^), performed counts of iron^+^CD68^+^ cells as a function of total CD68, and also counted the frequency of the iron^+^AIF1^+^CD206^+^ population expressing both general macrophage polarization markers selected in this study (Figures 3–3). The numbers of iron^+^ cells were less frequent than the general populations assessed above. In control tumors, 0.41% were iron^+^, 0.40% of AIF1^+^ were iron^+^, 0.61% of CD206^+^ TAMs were iron^+^, and 0.60% were iron^+^AIF1^+^CD206^+^ cells. Injection of USPIO led to significant increases in all these macrophage subsets with 4.9% iron^+^CD68^+^ cells (*p* < 0.01), 4.1% iron^+^AIF1^+^ (*p* < 0.01), 2.5% iron^+^CD206^+^ (*p* < 0.01), and 3.7% iron^+^AIF1^+^CD206^+^ cells observed after USPIO injection (*p* < 0.05). This indicates that SPIO injection increases iron in all polarized macrophage subsets but also indicates that inflammatory subsets take up relatively, but not significantly (*p* > 0.05), more iron than M2-like populations.

## 3. Discussion

The main purpose of the current work was to evaluate a computer vision method for detection and quantification of USPIO-enhanced macrophages by MRI and extend this analysis to histological images in order to characterize sub-MRI pixel phenotypes of the cells. By targeting the spatial heterogeneity in iron-based pixel contrast arising from endogenous or iron contrast agent-enhanced cellular iron deposits, this approach improved statistical quantification of macrophages over conventional ROI-average distribution analysis, and provided measured constraints on the size and frequency of the polarized macrophage deposits.

The MRI analysis approach presented here improves on current conventional ROI-based approaches by parsing the spatial distributions of iron image contrast. This was accomplished by constructing parametric iron MRI maps and then quantifying the number and size of pixel cluster areas as a function of stratified iron concentration range. This indicated that most of the area of tumor pixels in both control and nanoparticle-injected groups were predominantly of low iron contrast, reflecting a cellular distribution characteristic of the location of low-iron cancer cells and stroma in the tumor. The ROI distribution analysis only revealed minor shifts towards high iron concentrations with USPIO injection, while counting of the localized iron clusters increased the statistical differences between the control and USPIO-injected groups. A similar approach was used to evaluate the size of macrophage iron deposits in the histological analysis with and without contrast agents. This revealed areas of iron-laden macrophage colonies that were on the order of the size and frequency of the high-iron MRI clusters. This side-by-side spatial analysis confirmed the cellular sensitivity and specificity of the MRI and histological methods for detecting localized macrophage deposits according to the iron status, and provided per unit area cellularity estimates of the iron-laden macrophages detected in vivo by MRI.

To complement the iron histology analysis, we also performed immunofluorescent imaging focusing on the microenvironment and polarization characteristics of both general macrophage populations and specifically the iron^+^ macrophage subpopulations. The polarization of macrophages is a multifactorial process that depends on the tissue and microenvironment in which they are found as well as complex signaling between tissue resident and infiltrating immune cells with the macrophages [31, 32]. As such, myriad intracellular and surface protein markers have been developed that allow one to specify their position along the continuum of accessible polarization states [33]. Iron itself is a central metabolic factor in macrophage function and is associated with many polarization states [21]. To determine the association of iron deposits with the tumor microenvironment and polarization, we adopted a panel of general tissue and macrophage biomarkers which showed the iron^+^ macrophages were predominantly found in vascularized CD31^+^ regions of the stroma where they likely serve iron-handling functions in heme homeostasis [34–36]. Overall, these iron^+^ macrophages were found as a subpopulation of the total macrophages detected in the whole cross sections of stained tissues. Characterization of these general populations indicated that frequency of CD68^+^ macrophage infiltrates remained approximately unchanged with USPIO injection and further confirmed previous studies indicating that phenotypic inflammatory M1-like macrophage markers are increased and protumor M2-like phenotypes decreased with USPIO injection.

In our spatial analysis, we further characterized effects of USPIO on polarized endogenous and nanoparticle-enhanced iron^+^ macrophage deposits. We observed that USPIO injections increase iron in pan-macrophage CD68^+^ populations as expected and also found relative differences in frequency of iron-laden populations following USPIO injection in polarized subsets. Here, although inflammatory (AIF1^+^), wound-healing (CD206^+^), and double-positive macrophage populations (AIF1^+^CD206^+^) all increased frequency with USPIO injection, inflammatory macrophage populations experienced the largest relative increases presumably due to their predominant role in iron scavenging during the acute inflammatory response caused by the iron nanoparticles [22–24]. Therefore, spatial histological segmentation approaches based on iron status combined with macrophage polarization measurements in these regions allow for the further characterization of subpixel phenotypes of the iron-laden macrophage giving rise to the contrast measured by MRI. This provides further insight into the biological function of the macrophages detected and reveals differences in their iron handling roles in the tumor microenvironment.

## 4. Conclusions

While the spatial image analysis approach described here is based on identification of the macrophage according to the iron status, similar machine-based analyses are envisioned to be conducted utilizing other parametric MRI contrasts and other multimodality imaging contrasts. Further, in the current work, the approach is specific for the macrophage due to their high innate capacity for iron uptake; however, integrating other endogenous and contrast-agent enhanced parameters with these analysis tools can also potentially provide spatial information about different cellular populations in diverse tissues settings. We anticipate that the current findings will motivate the use of computer-assisted image analysis routines and accelerate the translation of these methods towards the clinic to aid in our imaging investigations of complex cellular microenvironments and physiological processes in diseases such as cancer.

## 5. Methods

### 5.1. Animal Procedures

All animal work was approved and performed according to the guidelines of the Animal Care and Use Committee of MSKCC. Mice were anesthetized with 1–3% isoflurane in O_2_ gas, and respiration was monitored during all imaging sessions. Female 6-week-old FVB/N mice underwent orthotopic injection into the lower mammary fat pad of 1 × 10^6^ syngeneic TS1 MMTV-PyMT tumor cells grown under standard tissue culture conditions and suspended in 100 *μ*L 50% Matrigel (BD Bioscience). Mice bearing orthotopic TS1 MMTV-PyMT tumors (approximately 1 cm^3^) were injected with either 0.5 mmol/kg dextran coated superparamagnetic 5 nm iron oxide nanoparticle contrast agent (Ocean NanoTech) or saline and were imaged 24 hr after injection.

### 5.2. MRI

^1^H MRI was conducted on a 7T/30 cm horizontal bore Biospec MRI system (Bruker BioSpin Corporation) with a custom-built 30 mm inner-diameter transmit-receive quadrature coil. A 2D multigradient echo (MGE) relaxometry pulse sequence with fat suppression was used with the following parameters: 16 evenly spaced 3 ms TEs, TR 1.2 s, matrix 256 × 256 in the plane data matrix with 25–49 slices, an in-plane spatial resolution of 0.1 mm × 0.1 mm with a slice thickness of 0.5 mm, and RF flip angle of 90°. Each phase encode acquisition was gated on the animal's respiratory cycle. The first image of the gradient-echo series was used as reference images shown in the figures.

Aqueous solutions of Fe^3+^(NO_3_^−^)_3_ (Fisher Scientific) were used as reference iron concentrations over the 0.0–0.3 mg iron(III) g^−1^ range at 7T [29]. The *T*_2_^*∗*^ values for these solutions were determined by pixel-wise monoexponential fitting of the MGE images using Matlab (Mathworks) and/or Fiji [37]. A linear relation between the relaxation rate *R*_2_^*∗*^=1/*T*_2_^*∗*^ and known iron concentration was found and was subsequently used to generate parametric iron MRI maps. Iron MRI maps were stratified by high concentration (total range, 0.0–0.3 mg·g^−1^; high, 0.15–0.3 mg·g^−1^). Spatial characteristics of the high-iron pixels were then quantified by performing cluster analysis over whole tissue MRI cross sections with the Fiji Analyze Cluster tool.

### 5.3. Histology

Whole tissue cross sections were sliced from the axial midpoint regions of PBS-perfused tumors following MRI studies, fixed in 4% PFA for 24 hours at 4°C, and then washed with H_2_O and resuspended in 70% ethanol (Fisher Scientific). Tissues were paraffin embedded, cut into 5 *μ*m sections, and placed on glass slides for immunohistochemistry.

The Prussian blue histochemical detection of iron(III) was performed by manually deparaffinizing in xylene and rehydration in series of alcohol dilutions (100%, 95%, and 70%) and tap water. Slides were then placed in a working solution of equal parts of 10% potassium ferricyanide (Fisher Scientific) and 10% hydrochloric acid (Fisher Scientific) prepared in distilled water and stained for 30 minutes. Slides were rinsed in distilled water, counterstained with nuclear-fast red, and cover slipped with Permount (Fisher Scientific).

The immunofluorescent detection of F4/80, CD31, CD68, AIF1, and CD206 was performed using a Discovery XT processor (Ventana Medical Systems). The tissue sections were deparaffinized with EZPrep buffer (Ventana Medical Systems), antigen retrieval was performed with the CC1 buffer (Ventana Medical Systems), and sections were blocked for 30 minutes with Background Buster solution (Innovex) followed by avidin/biotin blocking for 8 minutes. F4/80 (Abcam, cat# ab6640, 5 *µ*g/ml), CD31 (Dianova, cat# DIA-310 1 *µ*g/ml), CD68 (Boster, cat# PA1518, 5 *µ*g/ml), AIF1(Wako, cat# 019–19741, 0.5 *µ*g/ml), and CD206 (Abcam, cat# ab64693, 1 *µ*g/ml) were applied, and sections were incubated for 5 hours, followed by 60 minutes incubation with biotinylated goat anti-rabbit antibodies (Vector Labs, cat# PK6101) at 1 : 200 dilution. The detection was performed with Streptavidin-HRP D (part of the DABMap kit, Ventana Medical Systems), followed by incubation with Tyramide Alexa Fluor A488 (Invitrogen, cat# T20922), Tyramide Alexa Fluor 568 (Invitrogen, cat# T20948), or Tyramide Alexa 647 (Invitrogen, cat# B40958), respectively, prepared according to manufacturer's instructions with predetermined dilutions. After staining, slides were counterstained with DAPI (Sigma-Aldrich, cat# D9542, 5 *µ*g/ml) for 10 min and coverslipped with Mowiol. Histological sections were digitized with a Mirax Scan system and read with Panoramic Viewer (3DHISTECH, Budapest, Hungary). Images were first visually inspected, and then the whole images were exported and processed in Fiji. Iron deposits were quantified as described by Leftin et al. [29]. Briefly, iron deposit maps were generated by resizing the histological images by using pixel averaging and bilinear interpolation to down-sample the image size (1 : 100) to the resolution of the MRI experiment. The resulting masks of regions containing iron^+^ macrophages were discretized by watershed gradient processing, and spatial characteristics of the clusters were determined using the Fiji Analyze Cluster tool. The number of iron^+^ macrophages per cluster was then determined by using the cluster maps to define regions of interest for cell counts in the full-resolution histological images. Polarization state was determined by either exhaustive whole cross section counting of AIF1 or CD206 immunostained macrophages or localized analysis that was restricted to colocalized iron^+^ macrophages contained in the iron deposit regions.

### 5.4. Statistics

Two-tailed Student's *t*-tests were performed with significance determined as *p* < 0.05. All statistical calculations indicated in the text were performed with the GraphPad Prism 7 Software.

## Data Availability

All supporting data are found in the manuscript.
